# The digital keystone: how artificial intelligence is reshaping HLA research and clinical practice

**DOI:** 10.1007/s00251-026-01397-z

**Published:** 2026-04-27

**Authors:** Gamze Sonmez, Yigit Yazarkan, Deniz Cagdas

**Affiliations:** 1 Hacettepe University Faculty of Medicine, Ankara, Turkey; 2 Division of Pediatric Immunology, Department of Pediatrics, Faculty of Medicine, Hacettepe University, Ankara, Turkey; 3 Ihsan Dogramaci Childrens Hospital, Faculty of Medicine, Hacettepe University, Ankara, Turkey; 4 Department of Pediatric Immunology, Pediatric Basic Sciences, Institute of Child Health, Hacettepe University, Ankara, Turkey

**Keywords:** HLA, Machine learning, Deep learning, Allele-specific expression, Loss of heterozygosity

## Abstract

The human leukocyte antigen (HLA) system underpins allorecognition and shapes the response to infection, autoimmunity, and treatment response. Technological advances from serology to next-generation sequencing now enable full-gene characterization and four-field HLA nomenclature, while artificial intelligence (AI) and machine learning are transforming the data generation, interpretation, and clinical use. This review summarizes the progress on the technical developments in the HLA era, which could be evaluated in three perspectives. First, we survey AI for antigen processing and T-cell recognition, including HLA–peptide binding, presentation, and T cell receptor (TCR)–epitope models, and outline their effects on applications like neoantigen discovery, vaccine design, and tolerance induction. Since there are still persistent gaps in immunogenicity prediction and coverage of rare alleles, secondly, we evaluated HLA imputation from the single nucleotide polymorphism (SNP) arrays and low-coverage whole-genome sequencing, highlighting deep learning models that improve accuracy for common and low-frequency alleles, and the critical role of diverse reference panels. Third, we assessed the AI-enabled transplant decision support: survival and graft-versus-host disease forecasting from registry data, donor ranking beyond simple allele match, and crossmatch compatibility prediction. We integrate emerging biology, non-classical HLA molecules, allele-specific expression, and HLA loss of heterozygosity, as key modulators of immune activation and evasion with implications for donor selection, infectious diseases, vaccinology, inflammatory disease, and cancer therapy. To accelerate safe clinical translation, we need to have standards for data governance, fairness auditing, validation and calibration, explainability, robustness, monitoring, and human oversight. By bridging core HLA principles with recent biological insights and AI innovations, we outline a path toward reproducible and equitable clinical translation to immunogenomics in transplantation, infectious, inflammatory, oncologic diseases, and precision vaccinology.

## Introduction

The human leukocyte antigen (HLA) system, which constitutes the major histocompatibility complex (MHC) in humans, is the cornerstone of allorecognition in transplantation. These molecules are the principal alloantigens governing graft rejection and tolerance, making their study essential for solid organ and hematopoietic stem cell transplantation (HSCT) (Bodmer 2023). Beyond transplantation, HLA alleles influence susceptibility and progression of autoimmune/inflammatory diseases, determine drug hypersensitivity risk, and inform personalized immunotherapeutic strategies.

HLA molecules are divided into two major classes with distinct structures and functions. Class I proteins (HLA-A, -B, -C) present short peptides (8–11 amino acids) to CD8⁺ T cells, while Class II proteins (HLA-DR, -DQ, -DP) present longer peptides (13–25 amino acids) to CD4⁺ T cells. Both classes exhibit extraordinary polymorphism, particularly within the peptide-binding domains. This diversity allows the immune system to recognize a vast repertoire of pathogens and altered self-peptides. Accurately HLA-matching of donors and recipients at the level of the specific amino acid (AA) sequence, is therefore critical for clinical success.

The methods for identifying HLA alleles have evolved substantially. Early serological and molecular methods resolved only broad antigen groups, whereas later PCR-based approaches, such as sequence-specific primers (SSP) and sequence-specific oligonucleotide hybridization (SSO), improved resolution but were often limited to selected exons. Sanger sequencing enabled sequence-based HLA typing but frequently suffered from incomplete exon coverage and phase ambiguity, limiting reliable two-field allele assignment. In contrast, next-generation sequencing (NGS) allows comprehensive gene coverage, improved phasing, and reduced ambiguity, supporting accurate allele-level typing and, where required, higher field resolution and detection of structural variation. This level of precision enables discrimination between alleles differing by even a single amino acid change with potential functional consequences (De Santis et al. 2020; Lozac’hmeur et al. 2024; Ozaki et al. 2014; Shiina et al. 2012; Smith et al. 2019).

In parallel with these genotyping advances, artificial intelligence (AI) and machine learning (ML) are transforming HLA research. AI-driven methods can nowpredict which peptides will be presented by specific HLA molecules, and model T-cell recognition (Chang and Wu 2024; Cook et al. 2021; Xu et al. 2024). Clinically, these tools are being developed to help select donors, predict transplant complications, and forecast patient outcomes, bringing sophisticated decision support closer to the bedside (Weimer and Newhall 2025).

**Fig. 1:**
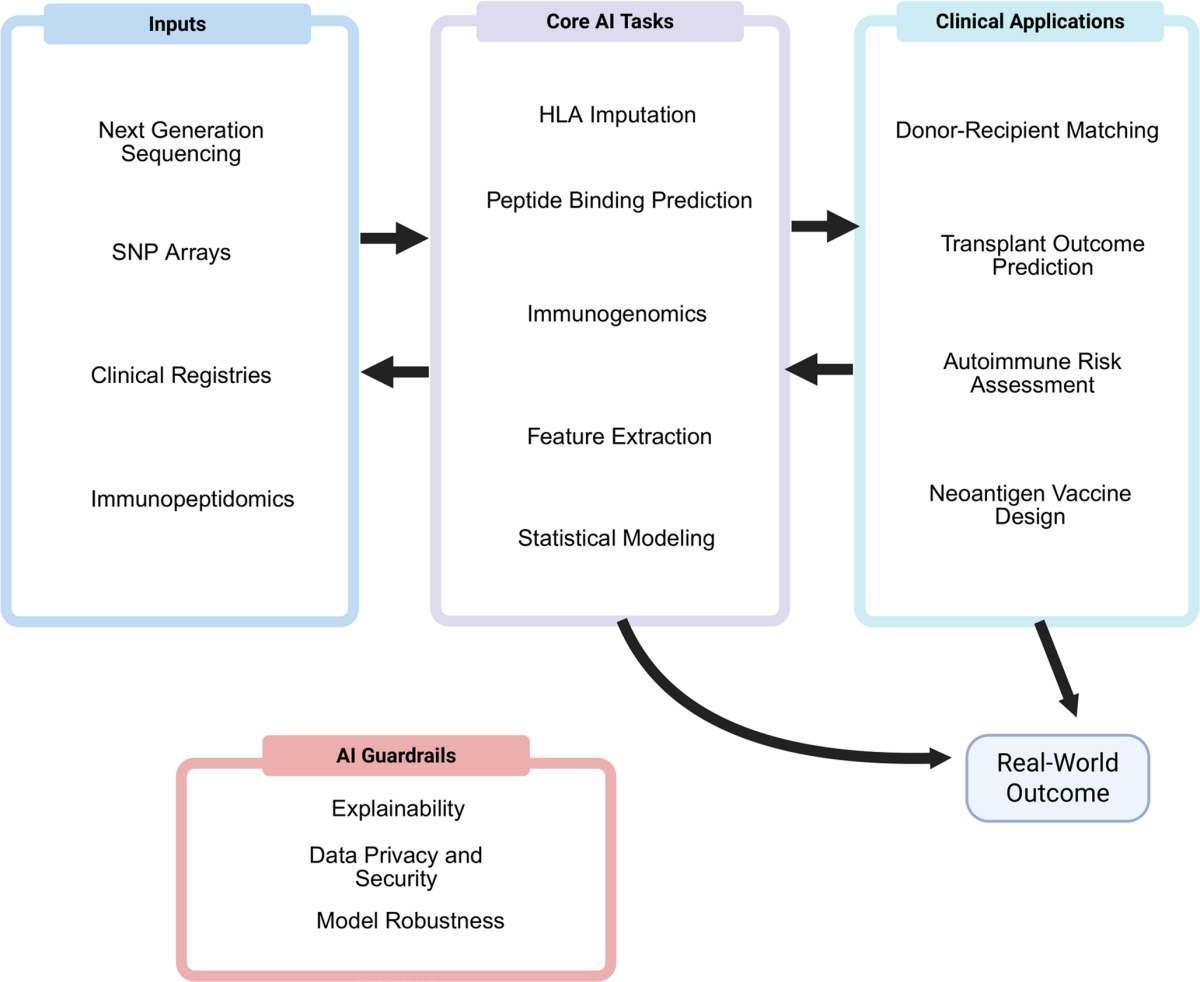
Data inputs—next-generation sequencing, SNP arrays, clinical registries, and immunopeptidomics—feed core AI tasks including HLA imputation, peptide–HLA binding/presentation prediction, immunogenomics, feature extraction, and statistical modeling. Model outputs drive clinical applications such as donor–recipient matching, transplant outcome prediction, autoimmune risk assessment, and neoantigen vaccine design. Arrows indicate information flow; bidirectional arrows denote iterative refinement between data and models. The lower pathway highlights real-world outcomes, which provide feedback to improve future models and datasets. AI guardrails (explainability, data privacy/security, and model robustness) apply across the pipeline to support safe, equitable deployment

However, the complexity of the HLA system extends beyond the genetic sequence. Emerging concepts, such as non-classical HLA molecules (HLA-E, -F, -G), allele-specific expression (ASE), and HLA loss of heterozygosity (LOH) reveal additional layers of immunoregulatory control and immune evasion (Lozac’hmeur et al. 2024; Wang and Greenland 2022). In this manuscript, we bridge core HLA principles with these recent biological insights and AI-enabled advances (Fig. 1). We aim to provide a coherent framework for current practice and delineate the standards needed to translate these powerful new tools into clinical implementation. While the term ‘AI’ is often applied broadly across information technologies, for the purposes of this review, we define AI specifically as the domain of ML and deep learning (DL) algorithms that learn patterns from data rather than following explicitly programmed rules.

## HLA imputation from SNP arrays and low-coverage whole genome sequencing

Since a high-resolution HLA typing at biobank scale is impractical, HLA is commonly imputed from single nucleotide polymorphism (SNP) data or low-coverage whole-genome sequencing (WGS) by exploiting linkage disequilibrium (LD) with alleles and training models on reference panels where SNPs and true HLA are both known. These approaches predict two-field (or higher) alleles accurately for many common variants (Table 1). Early LD-based frameworks, such as SNP2HLA, extended this paradigm by enabling simultaneous imputation of classical two and four-digit HLA alleles as well as amino acid polymorphisms within HLA genes, achieving around 95–97% accuracy for common four-digit alleles when trained on large European reference panels, demonstrating that reference panel size is a stronger determinant of accuracy than SNP density (Jia et al. 2013). Deep learning methods such as DEEP*HLA reach around 98–99% accuracy in European populations and show improved performance for low-frequency alleles in individuals with mixed ancestry and in populations that are less well represented in reference panels (Naito et al. 2021). Other ML models, including Support Vector Machines (SVMs) and Random Forest, can perform comparably to HLA Imputation with Attribute Bagging (HIBAG), a widely used method that infers HLA alleles from SNP data using ensemble classifiers trained on SNP–HLA haplotype relationships (Naito et al. 2021; Pappas et al. 2018). When trained on large, diverse reference panels, these approaches achieve similar accuracy. Pre-fit HIBAG reference panels for European, Asian, Hispanic, African, and multi-ethnic populations are publicly available through the HIBAG project website (Zheng et al. 2014). Accuracy depends strongly on allele frequency, panel size or diversity, and SNP density near HLA. Common alleles (> 5%) in well-represented groups often exceed 95% accuracy, whereas rare alleles (< 1%) or poorly represented populations can fall below 80% (Tanaka et al. 2024).

**Table 1:** Overview of key computational tools for HLA typing and imputation accuracy

Method/Tool	Approach	Input	Typical Resolution	Reported Accuracy	Notes	Reference
HIBAG (baseline)	Statistical/ensemble ML (reference panel–trained)	Genome-wide SNP array	Two-field or higher	> 95% for common alleles in Europeans	Performance depends on panel size and diversity	Zheng et al. 2014
SVM/ Random Forests	Supervised ML using LD between SNPs and HLA	Genome-wide SNP array	Two-field	Comparable to HIBAG with large diverse panels	Benefits from ethnically diverse references	Pappas et al. 2018
HLA*LA	Direct HLA typing from (low-coverage) WGS	Illumina WGS	High-resolution (gene to two-field)	99.4% average across six classical HLA genes on high-coverage Illumina WGS	Uniquely supports long-read data and assemblies	Dilthey et al. 2019
xHLA	Direct HLA typing from short-read WGS	Binary alignment and map of short-read NGS data	High-resolution	Reports 99–100% four-digit accuracy overall	Types six genes (HLA-A, -B, -C, -DQB1, -DRB1, -DPB1); the paper notes a dip in Class II exome accuracy likely due to exome capture pull-down bias	Xie et al. 2017
DEEP*HLA	DL trained on large WGS-derived panels	SNP array or low-coverage WGS	Two-field or higher	98–99% in Europeans; better for low-frequency alleles in mixed ancestry and in populations that are less well represented groups	Improved rare-allele imputation vs. classic methods	Naito et al. 2021
CookHLA	HLA genotype imputation from SNP arrays	GWAS SNP genotypes across the MHC (chr6) for target samples + a phased reference panel with SNPs and typed HLA alleles; alleles are binary-encoded markers.	Two-field resolution	Accuracy typically 97–98% in Europeans using large matched panels	Reference-panel dependent	Cook et al. 2021
HIBAG	Imputes HLA genotypes from SNP array data	Dense SNP genotypes and a phased reference panel	Four-digit (high-resolution)	High accuracy (92–98% for Europeans) with common arrays; provides posterior probabilities for QC.	Accuracy drops for rare alleles and under-represented ancestries	Zheng et al. 2014
SNP2HLA	HLA imputation from SNP arrays — imputes classical HLA alleles and amino-acid polymorphisms	GWAS SNP genotypes and a reference panel with SNP and HLA data	Four-digit (high-resolution)	Uses standard GWAS arrays; imputes both alleles and amino acids for fine-mapping; provides probabilistic dosages.	Population-specific; accuracy drops significantly with mismatched reference panels; cannot discover novel alleles.	Jia et al. 2013

*CNN* (convolutional neural network), *DL* (Deep Learning), *GWAS* (Genome-wide association studies), *HIBAG* (HLA Imputation with Attribute Bagging), *HLA* (human leukocyte antigen), *lcWGS* (low-coverage whole-genome sequencing), *LD* (linkage disequilibrium), *ML* (machine learning), *SNP* (single-nucleotide polymorphism), *SVM* (support vector machine), *WES* (whole-exome sequencing), *WGS* (whole-genome sequencing)

HLA imputation has revolutionized immunogenetics by enabling high-resolution MHC analysis within genome-wide association studies (GWAS). Beyond identifying susceptibility variants for autoimmune, infectious diseases and cancer (Kumar et al. 2014), this approach has defined essential pharmacogenomic associations- including B*57:01 with abacavir and B*15:02 with carbamazepine (Chang et al. 2023)- and transplant studies linking alleles or donor–recipient pairs to graft-versus-host disease (GvHD) or graft failure (Eapen et al. 2014; Petersdorf et al. 2020).

Because reference panels skew towards European or East Asian, efforts like HLA-Atlas (Marcu et al. 2021) and the International HLA and Immunogenetics Workshop (IHIW) aim to expand population diversity. In parallel, approaches that jointly impute HLA variation and expression quantitative trait loci are beginning to emerge (Middleton and Marsh 2013). As WGS costs drop, direct HLA typing with HLA*LA or xHLA increasingly outperforms SNP-only imputation, especially in diverse cohorts (Dilthey et al. 2019; Xie et al. 2017).

Deep learning approaches extend beyond accuracy improvements in typing and imputation. CookHLA introduces a hidden Markov model–based framework that embeds prediction markers into polymorphic HLA exons and adaptively learns population-specific genetic maps within the MHC, substantially reducing imputation error rates compared with SNP2HLA and HIBAG, particularly for rare alleles and in cross-ancestry settings (Cook et al. 2021).

DEEP*HLA, a convolutional neural network (CNN)-based framework trained on large WGS-derived reference panels, learns non-linear relationships between local SNP haplotypes and HLA alleles in a multi-task design, enabling more accurate imputation, including for low-frequency alleles in admixed or underrepresented populations, and supporting trans-ethnic fine-mapping of diseases, such as type 1 diabetes (Naito et al. 2021). In integrated workflows, SNP or WGS-based HLA inference can be paired with ligand prediction tools to not only recover high-resolution alleles but also prioritize their immunological relevance in infection, cancer, and transplantation.

Most current imputation frameworks have been developed and validated primarily for classical HLA loci, and their applicability to non-classical HLA genes remains limited. These loci exhibit lower polymorphism and are often underrepresented in reference panels, reducing imputation accuracy and reliability. As a result, non-classical HLA variation is less frequently captured in large-scale genetic studies. Emerging computational approaches therefore complement imputation by focusing on downstream functional prediction, such as peptide binding to non-classical HLA molecules, rather than high-resolution allele inference.

## Machine learning for HLA–peptide binding and T cell epitope prediction

### MHC binding and antigen presentation

Predicting which peptides bind to a given HLA allele is fundamental for understanding T cell immunity, vaccine design, cancer neoantigen discovery, autoimmune epitope mapping, and minor histocompatibility antigen discovery in HSCT. Classical motif-based methods relied on experimentally defined anchor residues but were limited by incomplete motif libraries. Over the past decade, sophisticated ML and DL models have been developed to improve binding affinity predictions and immunogenicity rankings (Doneva and Dimitrov 2024).

Early tools such as SYFPEITHI and BIMAS employ motif-based algorithms that score peptides based on known anchor residues (Larsen et al. 2005). While simple and interpretable, these methods suffer from incomplete motif coverage and poor performance for alleles lacking comprehensive experimental data. Position-specific scoring matrix (PSSM) methods, exemplified by NetMHC and NetMHCpan, combine quantitative binding data across peptide length and HLA alleles to generate allele-specific or pan-allele prediction models (Jurtz et al. 2017; Nielsen and Andreatta 2016). NetMHCpan (version 4.0) remains a widely used and reliable tool, with reported prediction accuracy measured by the area under the receiver operating characteristic curve (AUC) typically around 0.85–0.90 for peptide binding to HLA class I molecules (Jurtz et al. 2017). More recent versions further improve performance by integrating eluted ligand datasets and advanced ML frameworks that enable motif deconvolution and expanded allele coverage. Additionally, incorporation of structural features and transfer learning strategies has been shown to modestly enhance epitope prediction while maintaining broad applicability across diverse HLA alleles (Nilsson et al. 2025; Reynisson et al. 2020). In contrast to allele typing approaches, DeepHLAPred focuses specifically on peptide presentation, using DL to predict ligands for non-classical HLA class I molecules, which are often incompletely resolved by conventional typing pipelines (Huang et al. 2023). Similarly, HLAncPred applies ML–based classifiers to predict peptide binders for non-classical HLA class I alleles, particularly HLA-E and HLA-G, and has been used to prioritize viral epitopes, including candidates from SARS-CoV-2 variants (Dhall et al. 2022).

Table 2 summarizes key binding prediction tools, outlining their primary strengths and limitations.

**Table 2:** Summary of key AI and machine learning models in peptide-MHC binding and antigen presentation

Tool	Task	Typical inputs	Model family	Outputs	Common metrics	Strengths	Limitations	Reference
PLS-based NCAA–HLA-A*02:01 affinity predictor	Predicts binding affinity of peptides with non-canonical amino acids to HLA-A*02:01	Peptide sequences converted to physico-chemical vectors via SMILES strings, RDKit, and PCA	Classical ML: Partial Least Squares (PLS) regression, 3 components, 5-fold CV; benchmarked 36 regressors (e.g., ExtraTrees, GradientBoosting, Tweedie, SVR) via LazyPredict	Predicted log10 (IC50) (continuous); usable for affinity ranking and thresholding	Cross-validated (5-fold) R² = 0.477, RMSE = 0.735 (PLS best setting); per-fold tables compare PLS vs. top regressors	Explicitly handles NCAAs; fast structure-feature workflow; minimal MHC input (single allele); open-source code/data	Single-allele (A*02:01) and small dataset (~ 166 samples) cause variability; relies on accurate feature generation	Jiang et al. 2025
MUNIS	Predict HLA-I presentation and prioritize immunogenic CD8⁺ T-cell epitopes and immunodominant targets in pathogens; validated on EBV	HLA-I sequence/allele (α1/α2) + peptide (8–15 aa), optionally with ± 5-aa protein flanks; used to scan viral proteomes	DL	Presentation probability (0–1) and rank/%Rank; supports epitope prioritization and immunodominance ranking	Primary Average Precision (PR-AUC); also ROC-AUC;	Beats MixMHCpred/NetMHCpan/MHCflurry/TransPHLA/BigMHC on EL; strict train/test split; fewer false-positive binders (better motifs); finds novel EBV epitopes	Coverage limited to 205 HLA-I alleles; immunogenicity discrimination modest when conditioned on binders; no TCR-level modeling yet	Wohlwend et al. 2025
CapHLA	Peptide–HLA binding & presentation prediction	Peptide sequence (7–25 aa), HLA pseudo-sequence	Convolutional + multi-head self-attention neural network	Presentation probability and binding affinity	Evaluated with AUROC/AUPRC, accuracy, F1,	Unified model for both class I and II and variable peptide lengths	Neoantigen quality model currently validated mainly in HLA-I context; HLA-II immunogenicity less explored	Chang and Wu 2024
ImmuneApp	HLA-I antigen presentation, immunopeptidome deconvolution, neoepitope immunogenicity	Peptide sequence (8–15 aa) and multi-allelic immunopeptidomics samples	CNN + LSTM + attention “hybrid” DL; mono-allelic EL model	EL likelihood and presentation score and neoepitope immunogenicity score	AUROC, AUPRC, precision, recall, F1-score, and specificity for antigen-presentation prediction	Trained on large mono-allelic MS dataset plus deconvoluted multi-allelic data	Currently limited to HLA-I; HLA-II extension is planned but not yet available. Immunogenicity model mainly focuses on presentation; other determinants (processing, TCR recognition, tumor context) are only partially modeled.	Xu et al. 2024
Graph-pMHC	HLA class II antigen presentation prediction	Eluted ligand peptides (9–30 aa) with HLA-DR/DP/DQ sequences; uses AlphaFold2-derived structural data	Graph Neural Network using protein structure (AF2-based edges) as an inductive bias	Presentation score and predicted binding core position per peptide-allele pair	Average Precision (AP) / PR-AUC for presentation; on authors’ test set Graph-pMHC ≈ 81% AP vs. NetMHCIIpan-4.0 ≈ 61% ( ≈ + 20% absolute AP). ROC-AUC for antibody ADA risk	Strong structural inductive bias improves performance (+ 20% AP vs. NetMHCIIpan-4.0); handles flanking regions well; open-source	Relies on AlphaFold2 structural predictions, which can be imperfect; less interpretable; modest gains on clinical data	Thrift et al. 2024
DeepHLAPred	Predicts peptide binders for non-classical HLA Class I molecules (HLA-E and HLA-G)	Peptide sequence (8–15 aa) and a target HLA-E/G allele	DL (hybrid architecture): parallel CNNs + Bi-LSTM, with embedding and fully connected layers	Probability score (0–1) indicating binding likelihood; threshold-based classification into binder and non-binder	Sensitivity, Specificity, Accuracy, Matthews Correlation Coefficient, ROC-AUC	High predictive performance Captures both local (CNN) and sequential dependencies Combines multiple feature representations Works across multiple non-classical HLA alleles	Limited interpretability Performance depends on training dataset size and quality Focused only on non-classical HLA class I	Huang et al. 2023
HLAncPred	Prediction of non-classical HLA class I peptide binding	Peptide sequences (8–15 amino acids), encoded using binary profiles derived from amino acid composition and positional features	Classical ML models	Binary classification and prediction score; also supports promiscuous binding prediction across alleles	Sensitivity, Specificity, Accuracy	High performance Handles multiple alleles Interpretable feature design	Relies on engineered features Negative samples are synthetically generated, which may introduce bias Performance varies across alleles	Dhall et al. 2022
CapsNet-MHC	HLA class I peptide–MHC binding prediction	Peptide (8–14 aa) and HLA class I pseudo-sequences, encoded with BLOSUM62	Capsule Neural Network (CapsNet) that fuses peptide and HLA features via dynamic routing	Binary binding score/probability per peptide-allele pair	ROC-AUC, Spearman	Captures interaction patterns effectively; reports higher AUC than many baselines; robust with small datasets.	Predicts binding, not full antigen presentation; class I only; inherits biases from training data.	Kalemati et al. 2023
NetMHCIIpan-4.2	Predicts HLA class II antigen presentation	Large eluted ligand (EL) and binding affinity (BA) datasets, with new HLA-DQ-specific ligand data	Neural network ensemble (NNAlign_MA) trained on single- and multi-allelic data to deconvolve motifs	Presentation score (%Rank) and a prediction reliability score	AUC, PPV, and motif consistency	Greatly improves predictive power for HLA-DQ alleles; reduces incorrect assignments in multi-allelic data	Findings may be biased by training data composition; HLA-DQ prediction accuracy still lags behind HLA-DR for data-scarce alleles.	Nilsson et al. 2025
CAPTAn	Predicts HLA-II peptide ligands and prioritizes antigenic regions from source proteins	Peptide sequence (≤ 50 aa) + target HLA-II allele (core model); full source-protein sequence (context model); trained on mono-allelic EL-MS immunopeptidomes	DL: CNN motif detectors (core) + bi-LSTM context model; allele-specific ensemble weighting of core/context predictions	Per-allele ligand confidence/score and ranking; top-N epitope prioritization over proteomes; calibrated confidence bands	AUC-PR = 0.60, AUC-ROC = 0.83 (CV)	Expands DQ/DP coverage; learns contextual features from protein sequence; enabled discovery of microbiome and SARS-CoV-2 epitopes	HLA-II only; trained on potentially biased cell-line data; context model is not allele-specific	Strazar et al. 2023
NetMHCpan-4.1	Prediction of binding between peptides and MHC-I	Peptide list and target HLA allele(s) or sequence.	Neural network integrating binding affinity (BA) and eluted ligand (EL) data.	EL score and %Rank; optional BA prediction; binder calls.	FRANK score for epitope ranking.	State-of-the-art performance; pan-allele coverage.	Biased by MS artifacts in EL data; less accurate for epitope vs. ligand prediction; struggles with data-sparse alleles	Reynisson et al. 2020
NetMHCIIpan-4.0	prediction of binding between peptides and MHC-II	Peptides (typically length 15, user-set single length); selected DR/DQ/DP allele combinations	Artificial neural networks with NNAlign_MA to integrate BA + EL and deconvolute MA class-II peptidomes during training	Score_EL and %Rank_EL (default report); optional BA; %Rank-based SB/WB calls; reports predicted binding core	FRANK for CD4 epitope benchmarks; also AUC on peptide-sets constructed from source proteins.	Concurrent use of BA + EL and motif deconvolution improves class-II predictions over prior versions and competitors; broad allele support; pan-allele capability via sequence input	Performance can drop when adding context (observed in their tests); lower accuracy for alleles with few ligands (e.g., some HLA-DQ**);** inherits MS EL biases	Reynisson et al. 2020
NetMHCpan-3.0	Predicts peptide-MHC Class I binding affinity, modeling variable peptide lengths.	Peptide sequences (8–11 aa), MHC pseudo-sequence, and IC50 binding affinities from IEDB	Neural network ensemble (NNAlign) with a gapped alignment to handle variable lengths.	Affinity score, %Rank, and predicted binding core position.	PCC, ROC-AUC.	More accurate than fixed-length models; captures allele-specific length preferences	Predicts binding affinity, not antigen presentation; inherits training data biases	Nielsen and Andreatta 2016

*AA* (Amino Acid), *ANN* (Artificial Neural Network), *AP* (Average Precision), *AUROC/AUC* (Area Under the Receiver Operating Characteristic Curve), *BA* (Binding Affinity), *BLOSUM62* (Blocks Substitution Matrix 62), *CapsNet* (Capsule Network), *CNN* (Convolutional Neural Network), *CV* (Cross-Validation), *DL* (Deep Learning), *EL* (Eluted Ligand), *EL-MS* (Eluted Ligand Mass Spectrometry), *HLA* (Human Leukocyte Antigen), *HMM* (Hidden Markov Model), *IC50* (Half Maximal Inhibitory Concentration), *LSTM* (Long Short-Term Memory), *MA* (Multi-Allelic), *MARIA* (Multimodal Antigen pResentation wIth expression dAta), *MHC* (Major Histocompatibility Complex), *MS* (Mass Spectrometry), *MSIntrinsicEC* (Mass Spectrometry Intrinsic with Expression and Cleavage), *NCAA* (Non-Canonical Amino Acid), NetMHC/NetMHCpan/NetMHCIIpan (Neural Network–based MHC binding and presentation predictors), *NNAlign/NNAlign*_*MA* (Neural Network Alignment, Multi-Allelic), *PCA* (Principal Component Analysis), *PCC* (Pearson Correlation Coefficient), *PLS* (Partial Least Squares), *PR-AUC* (Precision–Recall Area Under Curve), *PSSM* (Position-Specific Scoring Matrix), *PPV* (Positive Predictive Value), *QM* (Quantitative Matrix), *RMSE* (Root Mean Squared Error), *ROC-AUC* (Receiver Operating Characteristic – Area Under Curve), *SB/WB* (Strong Binder/Weak Binder), *SMILES* (Simplified Molecular-Input Line-Entry System), *SMM* (Stabilized Matrix Method), *SRCC* (Spearman Rank Correlation Coefficient), *SVR* (Support Vector Regression), and *TCR* (T-cell Receptor)

#### **Chemistry-aware ML for non-canonical amino acids (NCAAs)**

A chemistry-aware ML approach predicts HLA-A*02:01 binding for peptides that include non-canonical amino acids (NCAAs) (Jiang et al. 2025). The method enables NCAA-aware affinity prediction without docking or structural inputs, with an accompanying implementation made publicly available by the authors. Key limitations are its focus on a single allele and the limited number of NCAA measurements; expanding allele coverage and using ensembles are proposed next steps.

#### MUNIS for T cell epitope discovery

Using AI may be helpful in discovering T cell vaccines. A dataset of 651,237 unique HLA class I ligands was assembled, and MUNIS, a DL model, was introduced to predict which peptides are presented by HLA class I alleles (Wohlwend et al. 2025). MUNIS outperforms existing tools at both peptide-presentation prediction and ranking CD8⁺ T-cell immunodominance. When applied to Epstein–Barr virus proteins, it recovered known epitopes and discovered new ones, which were confirmed by in vitro HLA class I–peptide stability assays and T-cell immunogenicity tests.

#### ImmuneApp for antigen presentation and immunopeptidomics

ImmuneApp is an interpretable DL framework that predicts HLA class I antigen presentation using large-scale immunopeptidomics datasets and integrates peptide and HLA representations (Xu et al. 2024). It improves prediction performance compared to existing tools and supports additional tasks, such as immunopeptidomics deconvolution and neoepitope prioritization. However, it is currently limited to HLA class I and does not fully incorporate other determinants of immunogenicity, such as antigen processing or TCR recognition.

#### graph-pMHC for class II prediction

graph-pMHC is a graph neural-network model for predicting HLA class II peptide presentation (Thrift et al. 2024). graph-pMHC builds residue–residue graphs from AlphaFold2-Multimer and uses a simple graph-enumeration step to align peptides in the MHC groove.

#### CapsNet-MHC for class I binding

CapsNet-MHC is a capsule-network model that predicts peptide-MHC class I binding. It outperforms other methods and maintains high accuracy even with limited training data (Kalemati et al. 2023).

#### CAPTAn for context-aware antigen discovery

CAPTAn is a context-aware DL model that predicts HLA class II peptide antigens by combining binding affinity with information from the full source-protein sequence (Strazar et al. 2023). Using CAPTAn, prevalent microbiome-derived T-cell epitopes and a SARS-CoV-2 epitope conserved across variants were uncovered. CAPTAn and its companion datasets provide a useful resource for antigen discovery and for probing genetic links between HLA alleles and immunopathology.

### TCR–pMHC and Repertoire Modeling

Table 3 summarizes key TCR–pMHC and repertoire-modeling tools, outlining their core capabilities, applications, and limitations.

**Table 3:** Summary of key AI and machine learning models in predicting TCR–pMHC interaction and repertoire modeling

Tool	Task	Typical inputs	Model family	Outputs	Common metrics	Strengths	Limitations	Reference
MixTCRpred	Predict epitope-specific TCR–pMHC interactions from paired αβ sequences	Paired αβ TCR sequences,, V/J genes; epitope-specific training sets	Transformer encoder with learned embeddings and positional encodings applied to concatenated CDR1/2/3 of α and β; one model per epitope (pMHC-specific). A pan-epitope variant also encodes the peptide but is secondary in the paper.	For a given TCR–pMHC: interaction score and % rank. Used for QC of barcoded-multimer scTCR-seq and to identify epitope-specific α-chains in dual-α cells	ROC-AUC in 5-fold CV, leave-one-sample-out, and leave-one-study-out; external IMMREP22 benchmark (retrained on shared splits). Reports median AUCs	Strong epitope-specific performance with ≥ 50 known TCRs; robust across cohorts; often beats pretrained baselines; practical %Rank; aids 10x multimer QC; validates α-chain disambiguation in dual-α cells.	Limited generalization to unseen; performance tied to training set size/bias; negative sampling may introduce decoy bias	Croce et al. 2024
DeepTCR	Epitope-specific TCR modeling and repertoire classification	TCR α/β CDR3 sequences and V/D/J gene usage from various experimental sources (e.g., tetramer sorts, scRNA-seq	DL suite including a VAE for TCR featurization and supervised CNNs for sequence and repertoire classification	TCR embeddings, antigen specificity scores, repertoire-level predictions, and interpretable motifs	ROC-AUC, F1-score for classification; various metrics for clustering and repertoire-level tasks	Learns joint sequence and V/D/J representations; handles noisy single-cell data; provides interpretable motifs; open-source	Performance is limited by training set size and quality; poor generalization to unseen epitopes	Sidhom et al. 2021
TITAN	Predict TCR–pMHC binding from paired TCR/epitope sequences; optimized for unseen-TCR generalization; analysis-ready	TCR sequences; epitopes encoded either as AA or SMILES (atom-level) for transfer learning	Bimodal 1D-CNN + cross-attention (TCR/epitope); optional BindingDB pretraining; SMILES augmentation; baseline: Levenshtein K-NN.	Binding/specificity probability for a TCR–epitope pair; attention maps highlighting influential residues/atoms	ROC-AUC and balanced accuracy under 10-fold CV on (i) “TCR split” (unseen TCRs, seen epitopes) and (ii) “strict split” (unseen TCRs & epitopes); external test on McPAS-TCR (seen vs. unseen epitope sets)	Encodes both TCR and epitope; provides interpretable attention; code available (MIT)	Modest generalization to unseen epitopes Performance varies with negative sampling and data sparsity/imbalance;	Weber et al. 2021

10 × (10x Genomics single-cell sequencing platform), *AA* (Amino Acid), *AUC* (Area Under the Curve), *CDR* (Complementarity-Determining Region), *CNN* (Convolutional Neural Network), *CV* (Cross-Validation), *DL* (Deep Learning), F1-score (Harmonic mean of precision and recall), *IEDB* (Immune Epitope Database), *IMMREP*22 (International Immunogenomics Research Project 2022 benchmark), *K-NN* (K-Nearest Neighbors), *McPAS-TCR* (McGill Public Archive of TCR Sequences), *MIT* (Massachusetts Institute of Technology, license context), *pMHC* (peptide–Major Histocompatibility Complex), *QC* (Quality Control), *ROC-AUC* (Receiver Operating Characteristic – Area Under Curve), *scRNA-seq* (Single-cell RNA sequencing), *scTCR-seq* (Single-cell TCR sequencing), *SMILES* (Simplified Molecular-Input Line-Entry System), TCR (T-cell Receptor), *VAE* (Variational Autoencoder), and *VDJ* (Variable, Diversity, Joining gene segments)

#### MixTCRpred for TCR-epitope recognition

A curated set of 17,715 paired αβ TCRs mapped to dozens of HLA class I or II epitopes was used to build MixTCRpred, an epitope-specific predictor of TCR–pMHC recognition. The model accurately recovers TCRs for multiple viral and cancer targets and doubles as a quality control (QC) tool for multiplexed single-cell TCR-seq. Applied to COVID-19 patient repertoires, MixTCRpred reveals enrichment of clonotypes predicted to recognize an immunodominant SARS-CoV-2 epitope (Croce et al. 2024).

#### DeepTCR

DeepTCR is a DL toolkit for T-cell repertoire analyses (Sidhom et al. 2021). It learns sequence “concepts” such as motifs and positional patterns from CDR3 and related features to support tasks like antigen classification and cohort-level repertoire comparison.

#### TITAN

TITAN is a bimodal attention network that jointly encodes TCR sequences and their cognate peptide epitopes to predict specificity (Weber et al. 2021).

## AI models for transplantation

### Transplant-oriented workflows and resources

Table 4 summarizes transplant-oriented workflows and resources, with side-by-side comparisons of tool inputs, model architectures, outputs, strengths, and limitations across application areas.

**Table 4:** Summary of key AI and machine learning models in transplant risk, resources & other workflows

Tool	Task	Typical inputs	Model family	Outputs	Common metrics	Strengths	Limitations	Reference
DARA	Predict physical flow-cytometric crossmatch from HLA antibody features	DSA MFI, donor/recipient HLA (imputed if needed), cPRA, and inferred DQ/DP heterodimers	Classical ML: RF/XGBoost/Balanced RF/RUS/LogReg; 75/25 split; custom FN-weighted cost; f1-weighted threshold; SMOTE/undersampling for imbalance.	Probability of positive crossmatch (T-cell/B-cell); thresholded pos/neg decision; feature permutation importance and “what-if” DSA MFI effects	ROC-AUC (AUROC 0.975 headline; overall AUC 0.965–0.979), Sensitivity, Specificity, PPV, NPV, LR±.	Outperforms manual virtual crossmatch; high specificity and improved sensitivity; reduces unnecessary tests and cold ischemia time	Lower sensitivity in some cases; accuracy depends on imputation quality; based on single-center retrospective data	Weimer and Newhall 2025
Machine-learning post-processing filter for MS immunopeptidomics	Filter out false-positive neoantigen candidates and rescue true positives by predicting MS/MS spectral consistency; improves identification from clinical tumor samples	Per-peptide descriptors incl. MaxMascotIonScore, NetMHCpan-4.1 MinRank (HLA binding), AliphaticIndex, ClinPredSynRTRatio (retention-time deviation); plus additional recorded/predicted features	Random forest regression trained to predict MS2Norm (MS2 peaks normalized by length); 5-fold CV on train; independent test set; LOO-CV to score all candidates for Mascot comparison	Per-candidate PredMS2Norm score and ranking; used as a filter/priority list for validation and downstream screening	Improved ROC-AUC 0.77 vs. Mascot 0.65; PR-AUC 0.28 vs. 0.18; fewer screens needed to recover HCS	Post-processing (no pipeline overhaul); leverages HLA affinity, physicochemical and RT deviation info; raises positive-hit rate 1.5–2× across cutoffs; can rescue low-Mascot true peptides	Single-center, small cohort and peptide set; depends on Mascot-proposed sequences; performance tied to NetMHCpan accuracy (rare alleles) and SAB/RT reliability; focused on SNV-derived 8–12mers	Weimer and Newhall 2025
MHC Motif Atlas	Resource/database for MHC binding specificities	Ligandomes from > 500 alleles. For alleles lacking ligands, inputs are MHC binding-site residues (34 positions) to predict motifs/length distribution	Unsupervised motif deconvolution + ML for length distributions	Motifs (PWM/PPM); length distributions; web visualization	For ML benchmarking (motif/length predictions): leave-one-allele-out, leave-ligands-out, and leave-30-alleles-out CV; similarity measured by Euclidean distance between predicted and experimental PWMs/length distributions;	Comprehensive, interpretable atlas, downloadable, curated datasets; helpful links to structures and external predictors	Uneven allele coverage; quality depends on source datasets	Tadros et al. 2023
Snowflake	Calculates B-cell epitope mismatch risk by modeling the surface accessibility of amino acid mismatches.	Donor and recipient HLA Class I alleles; trained on PDB and AlphaFold structures with docked peptides	DL model (bidirectional LSTM/BRNN) that predicts per-residue solvent accessibility	Per-residue solvent accessibility and an overall epitope mismatch score.	Model loss, MSE/SE comparisons to other tools, structural variance (RMSDA)	Allele-specific surface modeling that accounts for peptide effects; expands coverage using AlphaFold; supports imputed HLA types	Class I only; relies on predicted structures; training data is biased toward HLA-A*02:01; requires clinical validation	Niemann et al. 2022
Longitudinal Survival Prediction After Allo-HCT	Predict overall survival (OS) at 100 days, 1 year, and 2 years post-allogeneic hematopoietic cell transplantation (allo-HCT)	Baseline and longitudinal clinical variables	Naïve Bayes (supervised ML for classification)	Probability of survival (alive or deceased) at 100-day, 1-year, and 2-year marks	AUC (100-day: 0.879, 1-year: 0.745, 2-year: 0.722), Kaplan-Meier plots, Sensitivity, Specificity	Incorporates both baseline and dynamic, longitudinal data; improves prediction accuracy over traditional risk scores	Retrospective design; short observation window (30 days post-transplant); limited generalizability for some patient groups	Zhou et al. 2024
Decision Tree / Random Forest model for antibody-incompatible kidney transplant rejection prediction	Predict early antibody-mediated rejection risk in kidney transplantation	Pre-transplant clinical and immunologic variables: highest IgG DSA MFI, IgG subclass levels (IgG1-4), number of HLA mismatches, cytometry crossmatch type, patient demographics, transplant characteristics (14 baseline features)	Classical ML: Decision Tree and Random Forest; small-data training with repeated resampling	Probability and classification of early rejection; interpretable decision rules identifying antibody thresholds	Accuracy, Sensitivity, Specificity, PPV, NPV, ROC-AUC	Interpretable thresholds for risk; works with small datasets; identifies key immunologic risk factors; supports clinical decision-making	Small single-center dataset ; risk of overfitting; limited generalizability;	Shaikhina et al. 2019
Mono-allelic LC-MS/MS HLA peptidome predictor	Predict endogenous HLA class I peptide presentation and improve epitope prediction by integrating mass-spectrometry-derived ligand data	Mono-allelic LC-MS/MS–identified peptides, peptide sequences, RNA-seq expression, proteasomal cleavage context, HLA allele identity, biochemical features	Neural networks (single-layer artificial networks) trained on mono-allelic MS datasets; integrative ML combining binding affinity, expression, and processing features	Probability/ranking of peptide presentation; predicted binding motifs; improved epitope prioritization lists	Positive Predictive Value (PPV), ROC-AUC (> 0.98), cross-validation performance; external dataset validation	Large unbiased MS-derived training set; improves prediction beyond affinity-only models; integrates biological processing steps (expression + cleavage); higher PPV	Requires mono-allelic MS datasets; mainly HLA class I; dependent on MS quality and allele coverage; computational pipeline complexity	Abelin, J.G. et al. 2017
ML-based neoantigen immunogenicity ranking framework	Predict and prioritize immunogenic neoantigens and mutations for cancer immunotherapy by integrating antigen presentation and T-cell recognition features	WES-derived mutations, RNA-seq expression, HLA binding predictions, peptide stability, proteasomal processing scores, binding promiscuity, oncogenicity annotations (IntOGen), immunopeptidome overlap (ipMSDB),	Classical ML ensemble: logistic regression, XGBoost, CatBoost, SVM; voting classifier combining LR + XGBoost; cross-validation (leave-one-out and external dataset validation)	Probability/ranking of immunogenic neo-peptides or mutations; prioritized candidate lists for vaccine or T-cell therapy design; feature importance via Shapley values	Fraction ranked, top-20 immunogenic fraction (TTIF), AUPRC, ROC-based evaluation; improved ranking	Integrates multi-dimensional biological features beyond binding affinity; harmonized datasets improve generalizability; strong cross-dataset performance; interpretable feature importance;	Dependent on available immunogenicity datasets; mostly SNV-derived peptides; potential bias from screening strategies; limited HLA class II modeling	Müller et al. 2023
DASH (Deletion of Allele-Specific HLAs)	Detect allele-specific HLA LOH from paired tumor-normal sequencing data to identify immune escape mechanisms	Paired tumor and normal WES data, patient-specific HLA typing, allele-specific sequencing depth, flanking region CNV signals, tumor purity	Classical ML: gradient-boosted decision trees (XGBoost) trained on engineered allele-specific genomic features with 10-fold cross-validation	Classification of allele-specific HLA LOH; probability of deletion; identification of lost allele; population-level LOH prevalence analysis	Sensitivity, Specificity, AUROC, AUPRC, F1 score; cross-validation and orthogonal digital PCR validation	High sensitivity to subclonal events; allele-specific detection; robust across tumor purity levels; validated with digital PCR; integrates genomic context features	Performance influenced by sequencing depth and platform; mainly HLA class I; requires paired tumor-normal data and high-quality HLA typing	Pyke et al. 2022

*AA* (Amino Acid), *ACC* (Accuracy), *AF* (Allele Frequency), *AUROC/AUC* (Area Under the Receiver Operating Characteristic Curve / Area Under the Curve), *BRNN* (Bidirectional Recurrent Neural Network), *CLI* (Command Line Interface), *cPRA* (Calculated Panel Reactive Antibody), *CV* (Cross-Validation), DSA (Donor-Specific Antibody), *FN* (False Negative), *HCS* (High-Confidence Set), *HLA* (Human Leukocyte Antigen), *LOH* (Loss of heterozygosity), LR± (Likelihood Ratio Positive/Negative), *LSTM* (Long Short-Term Memory), *MCC* (Matthews Correlation Coefficient), *MFI* (Mean Fluorescence Intensity), *MHC* (Major Histocompatibility Complex), *ML* (Machine Learning), *MS* (Mass Spectrometry), *MS2Norm* (Normalized MS/MS peak intensity), *MSE/SE* (Mean Squared Error / Standard Error), *NPV* (Negative Predictive Value), *PDB* (Protein Data Bank), *PPM/PWM* (Position Probability Matrix / Position Weight Matrix), *PPV* (Positive Predictive Value), *PR-AUC* (Precision–Recall Area Under Curve), *QC* (Quality Control), *RF* (Random Forest), *RMSDA* (Root-Mean-Square Deviation of Accessibility), *ROC-AUC* (Receiver Operating Characteristic – Area Under Curve), *RUS* (Random Under-Sampling), *SAB* (Single Antigen Bead), *SMOTE* (Synthetic Minority Over-sampling Technique), *SNV* (Single Nucleotide Variant), *SVC* (Support Vector Classifier), *T-cell*/*B-cell* (T lymphocyte / B lymphocyte), and UI (User Interface)

The `MHC Motif Atlas` is a critical resource for understanding immune responses in transplantation (Tadros et al. 2023). By consolidating allele-specific ligands, binding motifs, and length distributions across hundreds of HLA alleles, this tool plays a key role in predicting transplant outcomes. By predicting peptide-HLA interactions, it helps identify mismatches between donor and recipient, which can lead to graft rejection. The MHC Motif Atlas leverages ML to fill gaps in under-profiled alleles, offering insights that can improve the accuracy of transplant outcome predictions.

`Digital Alloimmune Risk Assessment` (DARA), for instance, predicts crossmatch (donor-specific antibody reactivity) outcomes using HLA antibody features, providing high specificity and sensitivity, which reduces unnecessary tests and ischemia time in solid organ transplantation (Weimer and Newhall 2025). `Snowflake` uses DL to model epitope mismatches based on surface accessibility, predicting the risk of B-cell epitope mismatch, which is crucial for transplant compatibility solid organ transplantation (Niemann et al. 2022).

Mass spectrometry (MS)-based immunopeptidomics, not an in-silico but a high-throughput technique, identifies the entire peptide repertoire naturally presented by HLA molecules on the cell surface. Machine-learning post-processing filters for MS immunopeptidomics can enhance neoantigen identification, improving transplant monitoring. These resources offer varied and powerful models, from classical ML- techniques like Random Forest and XGBoost to more advanced DL models, allowing for improvements in transplant risk prediction, immunological profiling, and patient-specific outcome forecasting.

These models may also be used in the treatment of other clinical situations, such as cancer therapy.

### AI-assisted donor selection and risk stratification

Artifical intelligance models can rank potential donors not solely by allele match but by integrated survival probability. For example, a 9/10 HLA match with a younger donor may yield a predicted 5-year overall survival comparable to a 10/10 match with an older donor (Weimer and Newhall 2025). Such insights enable transplant physicians to select donors who optimize long-term outcomes. Classifiers trained on historical transplant data can forecast a patient’s risk of grade II–IV acute GvHD based on donor–recipient HLA combinations, presence of preformed donor-specific antibodies, and conditioning regimen intensity, enabling preemptive adjustments to GvHD prophylaxis (Och et al. 2024). Predictive frameworks that incorporate early post-transplant biomarkers such as cytokine profiles, chimerism levels, and immune cell subsets, alongside baseline HLA mismatch data can detect impending GvHD or graft failure, allowing for timely interventions such as steroid adjustments, extracorporeal photopheresis, or donor lymphocyte infusion.

Weimer et al. developed and validated a DARA tool that applies machine learning to HLA antibody data to predict physical crossmatch compatibility (Weimer and Newhall 2025). Shaikhina et al. trained ML models on a small cohort of 80 kidney transplants using variables such as age, sex, HLA class I/II, dialysis duration, immunoglobulin (Ig) G subclass, and peak donor-specific antibody (DSA) (IgG) levels, and prior transplants; their best model predicted 30-day acute antibody-mediated rejection (AMR) with 85% accuracy (Shaikhina et al. 2019).

Abelin et al. introduced a scalable mono-allelic liquid chromatography tandem mass spectrometry (LC–MS/MS) pipeline to profile endogenous HLA class I peptidomes, engineering B721.221 cells to express one allele at a time and identifying more than 24,000 peptides across 16 alleles with stringent false discovery rate (FDR) control and minimal MS bias (typically 900–3,550 peptides per allele). This approach revealed subdominant binding motifs that are underrepresented in the Immune Epitope Database (IEDB) (Abelin et al. 2017). Building on these data, the authors quantified how binding affinity combines with gene/protein expression and proteasomal cleavage to shape presentation, and trained neural-network models that outperformed affinity-trained predictors, with positive predictive value rising when expression/cleavage features were added. Together, the mono-allelic MS approach and MS-trained models markedly improved epitope prediction over standard tools.

### Predicting survival and GvHD from registry data

Large transplant registries (e.g., NMDP, CIBMTR, Eurotransplant) collect comprehensive donor–recipient data, including high-resolution HLA genotypes, immunosuppression regimens, conditioning protocols, demographics, comorbidities, and longitudinal outcomes (overall survival, acute and chronic GvHD, relapse) (Guerrettaz et al. 2025). These “big data” resources have catalyzed the development of AI-driven predictive models aimed at optimizing donor selection, tailoring prophylaxis, and identifying patients at high risk for post-transplant complications (Thongprayoon et al. 2020).

Supervised learning models, such as Random Forest,, gradient boosting machines, such as XGBoost, LightGBM, and feedforward neural networks are trained on retrospective datasets to predict overall survival, nonrelapse mortality, grade II–IV acute GvHD, and chronic GvHD. Input features often include detailed HLA mismatch information, donor age, recipient comorbidities, conditioning intensity, and graft source. Many of these models achieve concordance indices (C-indices) of around 0.70–0.75 for overall survival prediction, matching or slightly exceeding the performance of traditional Cox proportional hazards regression (Zhou et al. 2024). More complex architectures, such as deep neural networks with embedding layers for categorical variables (including HLA alleles, donor–recipient race/ethnicity, and conditioning regimens) have demonstrated incremental performance gains but require large sample sizes to avoid overfitting.

### Models for AI-integration to clinical translation

Artificial intelligence models that combine high-resolution HLA genotypes with clinical and multi-omics data can turn immunogenomic insight into transplant decisions (Abelin et al. 2017; O’Donnell et al. 2020). Gradient-boosted tree models; including XGBoost-based predictors of acute antibody-mediated rejection using DSA features (Shaikhina et al. 2019) and survival or GvHD forecasting models trained on CIBMTR registry data (Zhou et al. 2024), integrate allele or AA-level HLA features, donor–recipient mismatch metrics, and clinical covariates to support donor ranking and risk stratification. NetMHCpan and mono-allelic immunopeptidomics further refine patient- and population-specific epitope maps by modeling binding, processing, and surface presentation. Safe deployment of these models requires independent calibration, decision-curve analysis, prospective multi-center validation, and fairness audits; future work should map HLA–pathogen relationships to better tailor prophylaxis, immune monitoring, and vaccines in transplant populations. Unlike traditional discrimination and calibration metrics, decision-curve analysis explicitly quantifies the clinical utility of a model by comparing the net benefit of using predictions to guide decisions at different threshold probabilities versus default strategies, such as “treat all” or “treat none” (Chalkou et al. 2023). This ensures that a model recommended for donor selection or GvHD prophylaxis truly leads to better patient outcomes given real-world risk-tolerance tradeoffs. Decision curve analysis is now widely recommended for evaluating clinical AI before deployment.

### Allele-specific HLA expression

In heterozygous individuals, the two alleles at a given HLA locus can be expressed at markedly different levels on the cell surface. Such allele-specific HLA expression (ASE) arises from cis‐regulatory variants in promoters, enhancers, or 3′ untranslated regions (UTRs), as well as epigenetic modifications that alter transcription or mRNA stability (Williams 2001). RNA-sequencing (RNA-seq) or targeted long-read transcriptomic approaches enable quantification of allele‐specific transcripts by counting diagnostic SNPs or capturing full-length HLA isoforms, while flow cytometry with allele-specific antibodies can measure protein level differences.

Clinically, ASE has important implications in both HSCT and solid organ transplantation. In HSCT, mismatches involving high-expressing alleles have been shown to drive stronger donor T cell responses and increase acute GvHD risk, whereas mismatches involving low-expressing alleles may be tolerated more readily, allowing “acceptable” mismatches at HLA-C or HLA-DP. Moreover, during the HSCT process, residual leukemic blasts can downregulate or entirely lose expression of a mismatched HLA allele, a form of ASE loss, enabling immune escape and contributing to relapse; early detection via chimerism assays can guide interventions, such as donor lymphocyte infusions (Hirabayashi et al. 2016).

In kidney transplantation, donors whose mismatched HLA alleles are naturally low expressing may provoke weaker alloantibody responses, translating into better long-term graft function despite allele-level mismatches (Kosmoliaptsis et al. 2009). Underlying ASE mechanisms include promoter or enhancer polymorphisms, inflammation-induced DNA methylation changes that transiently boost class I expression, and post-transcriptional control via microRNA binding sites in 3′ UTRs (Castelli et al. 2021).

Müller and colleagues trained ML classifiers to prioritize truly immunogenic tumor neoantigens using whole exome sequencing (WES) and RNA-seq data from 131 patients (Müller et al. 2023). This can be strengthened by integrating ASE. Because heterozygotes often express their two HLA alleles at different levels, peptides that (i) derive from transcripts demonstrably expressed in the tumor and (ii) are presented by higher-expressing HLA alleles should be up-weighted, while peptides tied to alleles with low or lost expression should be down-weighted. Adding these ASE signals to Müller and colleagues’s existing features should improve cross-dataset generalization and make the ranking of clinically actionable neoantigens more reliable.

### HLA loss of heterozygosity and immune escape

HLA LOH refers to the somatic loss or downregulation of one HLA allele, often via chromosomal copy-number loss, uniparental disomy, or epigenetic silencing, in tumor cells or allografts, representing a key mechanism of immune evasion (Lozac’hmeur et al. 2024). In cancer, LOH at the HLA locus removes the allele presenting dominant neoantigens, allowing tumor clones to escape T cell surveillance; WES or SNP array analyses can identify such LOH regions, while flow cytometry or allele-specific quantitative PCR can confirm loss of cell-surface expression. Many solid tumors, such as non–small cell lung cancer and melanoma, acquire HLA class I LOH at relapse, correlating with resistance to checkpoint inhibitors. Monitoring LOH thus has potential to guide therapeutic shifts, for example from PD-1 blockade to natural killer (NK) cell–based approaches that do not rely on classical HLA recognition (Perea et al. 2018).

In approximately 10–15% of relapsed acute myeloid leukemia (AML), leukemic blasts acquire LOH at mismatched HLA-A or HLA-C alleles, escaping donor graft-versus-leukemia effects; early detection using deep NGS–based chimerism assays can prompt alternative interventions, such as NK cell therapies, bispecific antibodies, or TCR engineering against non-lost antigens (Rovatti et al. 2020).

In solid organ transplantation, donor endothelial cells under intense immunosuppression can undergo somatic LOH of host-specific HLA alleles, which may contribute to operational tolerance, a state where the recipient maintains stable graft function without ongoing rejection even with reduced or no immunosuppression, leading to prolonged graft survival (Roussey-Kesler et al. 2006). Detection of HLA LOH in tumor biopsies may serve as a biomarker for acquired resistance to T cell–engaging therapies (for example, CAR-T cells targeting HLA-restricted targets), and routine WES or deep NGS of minimal residual disease samples could reveal emerging LOH events early, enabling timely shifts in therapeutic strategy.

Accurate identification of HLA LOH from sequencing data has become increasingly important as tumor molecular profiling becomes routine. Most copy-number callers first estimate tumor purity and ploidy, then model expected relationships among B-allele frequency (the read-depth ratio B/(A + B)), local sequencing depth, and deletion events. However, when these generic tools are applied to the HLA locus, performance is often unreliable: the extreme polymorphism of HLA causes mapping artifacts and misestimation, and the resulting calls rarely indicate which specific HLA allele is lost, the detail that is essential for neoantigen-focused therapies. Although the region has long posed analytical challenges, two advances, alignment to allele-specific references and the adoption of genome graph approaches, have improved HLA typing and somatic variant detection, setting the stage for more precise HLA-LOH inference.

For validating HLA-LOH callers, two strategies are commonly used. The first checks agreement between HLA-LOH calls and copy-number changes inferred by standard CNV tools in regions flanking each HLA gene. The second designs primers around the HLA loci and uses PCR to test for copy-number loss in cases predicted to involve all HLA loci. Yet neither approach confirms which specific allele is missing, nor do they rigorously assess performance in low-purity tumors or in samples with subclonal HLA-LOH, limiting their value as orthogonal evidence for clinical approval. Pyke and colleagues developed DASH (Deletion of Allele-Specific HLAs), a ML method tailored to the HLA locus that improves allele-specific LOH detection and defines limits of detection for subclonal events (Pyke et al. 2022).

## A practical framework for Tool selection and clinical translation

To translate AI or ML advances into clinically useful immunogenetic tooling, it is essential to match each analytical question with the most appropriate model class and data type. For HLA genotyping and imputation, DL approaches such as DEEP*HLA improve accuracy for low-frequency alleles when reference panels are large and ancestrally diverse, whereas ensemble statistical models such as HIBAG remain preferable when SNP array density or population representation is limited (Huang et al. 2024).

For antigen processing, MS-trained neural networks such as MUNIS and chemistry-aware models extend coverage beyond canonical class I molecules, while NetMHCpan remains the strongest option for pan-allelic inference when experimental ligand data are sparse (Jurtz et al. 2017). TCR-specificity frameworks must be selected based on data availability: attention-based models such as TITAN excel when paired TCR αβ data and high-quality epitope maps are present, while repertoire-level tools like DeepTCR scale more robustly to large, partially labeled single-cell datasets.

For outcome prediction in transplantation, gradient-boosted decision trees and neural networks with HLA embeddings can outperform Cox proportional hazards for donor ranking and GvHD prediction, but require careful calibration, external validation, and fairness auditing prior to clinical use.

Importantly, there is no universally optimal algorithm: performance is task-dependent, data-dependent, and population-dependent. Matching model class to (i) target allele frequency spectra, (ii) training cohort diversity, and (iii) intended clinical action is essential to ensure both accuracy and equity. This framework highlights where AI tools are already clinically robust (e.g., crossmatch prediction, mono-allelic MS–guided epitope prioritization), and where major bottlenecks remain, including limited non-European representation in reference panels, inconsistent benchmarking across pipelines, and limited demonstration of clinical net benefit. By explicitly aligning model choice with practical objectives, this framework provides a foundation for responsible deployment of HLA-aware AI in transplantation, immuno-oncology, and vaccine design.

## Conclusions and future directions

**Fig. 2:**
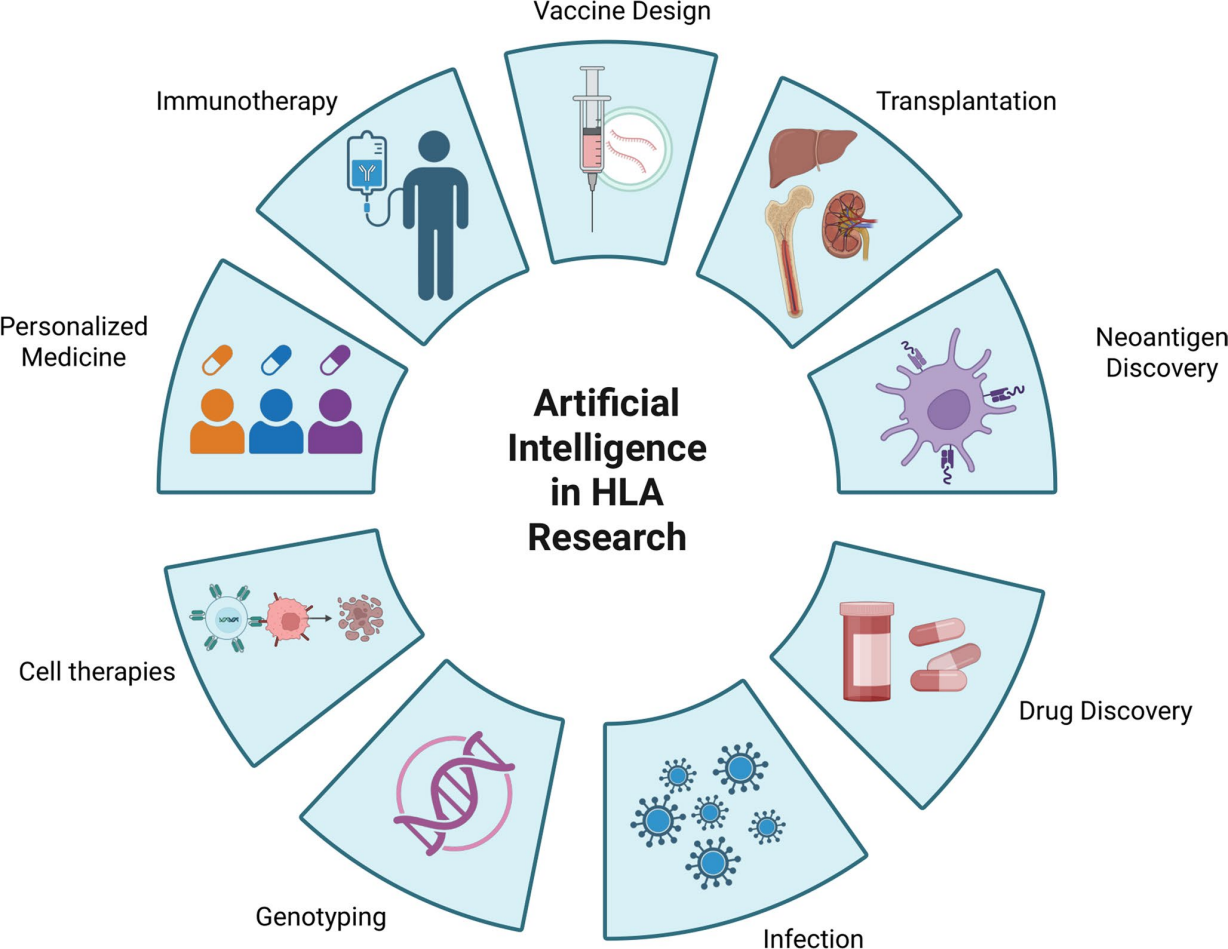
Applications of Artificial Intelligence in HLA Research. The figure illustrates the central role of AI in advancing key areas of HLA research and its clinical applications to provide precision medicine. These domains include transplantation, vaccine design, neoantigen discovery, immunotherapy, and personalized medicine

Over the past four decades, the field of immunogenetics has been defined by a dual revolution: the relentless advance of HLA typing technology from low-resolution serology to high-resolution NGS, and the concurrent rise of powerful computational tools. The progress in genotyping has improved graft survival in transplantation by enabling precise allele-level matching (Dehn et al. 2019). In parallel, AI and ML have amplified these gains, powering applications across the research pipeline: from imputing HLA genotypes in large biobanks and predicting peptide-HLA binding for vaccine design, to forecasting transplant outcomes and optimizing donor selection (Fig. 2). This synergy is further enriched by a deeper biological understanding of phenomena like ASE and LOH, which reveal how HLA expression is dynamically regulated and how cancers can evade T-cell surveillance.

A significant challenge in developing these models is that registry data is often heterogeneous, with different centers using varied protocols and having incomplete data. This can cause a model trained on one registry’s data to perform poorly on another’s. Federated learning, a method where models are trained across multiple decentralized datasets without sharing patient data, is a promising solution to improve generalizability while maintaining privacy. Another major hurdle is the “black box” nature of complex models, which can limit clinical adoption. The development of Explainable AI (XAI) is critical for building trust, allowing models to provide not just a prediction but also the key factors driving that prediction. Looking ahead, digital twins, dynamic, patient-specific models that simulate outcomes under alternative donors and regimens, are a compelling goal. Critically, all such tools require prospective, multi-center clinical validation before broad deployment.

Safe, equitable use of HLA-aware AI demands guardrails across data, modeling, and practice (Fig. 1). First, data governance must ensure appropriate consent, de-identification, and secure handling of genetic information, using privacy-preserving methods such as federated training when data cannot leave local institutions. Fairness should be routinely assessed by auditing discrimination and calibration across ancestry, sex, age, and allele-frequency strata, and by reporting clinically meaningful metrics such as decision-curve net benefit alongside AUC. Rigorous internal and external validation with prespecified endpoints is essential, including calibration checks, such as reliability diagrams or calibration belts. Transparency should be promoted through explainability tools (e.g., feature attributions) and through model cards and dataset statements that document intended use and limitations. Robustness must be stress-tested against label noise, class imbalance, missing data, and distribution shifts. Continuous monitoring should track data or performance drift, incorporate systematic versioning, and allow rollback of unsafe updates. Finally, strong human oversight is necessary, with clear definition of on and off-label populations and explicit clinical actions associated with model outputs.

Beyond these technical hurdles, the integration of AI into clinical practice raises significant ethical questions. A primary concern is algorithmic bias. The accuracy of HLA imputation and outcome prediction models is limited by the lack of comprehensive, ethnically diverse reference panels. Relying on models trained on skewed data risks creating or worsening health disparities for underrepresented populations. Furthermore, the use of sensitive genomic and clinical data demands robust data privacy and security protocols. Methodologies like federated learning offer a promising path forward. Finally, as these technologies mature, we must ensure equitable access to prevent a future where the benefits of AI-driven immunogenetics are available only to a select few.

On the other hand, in cancer immunotherapy, exome and transcriptome sequencing of tumors generate candidate neoantigen peptides, which are filtered through ML models for predicted binding to a patient’s specific HLA alleles. Top-scoring neoantigens, those with high predicted binding affinity and immunogenicity, are prioritized for personalized vaccine or adoptive T cell therapies. Early clinical trials in melanoma, glioblastoma, and non–small cell lung cancer have demonstrated safety and preliminary efficacy of neoantigen vaccines guided by these AI models (Li et al. 2024). In infectious disease vaccine development, AI-driven prediction accelerates identification of conserved epitopes across rapidly evolving viruses, such as influenza and SARS-CoV-2, optimizing peptide cocktails to maximize population coverage based on global HLA allele frequencies (Yarmarkovich et al. 2020). Prospective clinical validation is also needed: while retrospective and early-phase studies in neoantigen vaccine trials are promising, larger cohorts and long-term follow-up are essential to demonstrate true clinical utility (Freudenmann et al. 2018).

Looking forward, the future of HLA research lies in integration and personalization. Technologies like single-cell HLA sequencing combined with TCR-repertoire analysis promise to deliver an unparalleled view of alloimmunity and anti-tumor responses. The ultimate goal is to integrate high-resolution HLA data with other “omic” layers; transcriptomics, proteomics, and epigenomics to create dynamic, AI-driven models of each patient’s immune system. By responsibly navigating the technical and ethical challenges, we can harness these innovations to usher in a new era of personalized medicine, dramatically improving outcomes in transplantation, cancer immunotherapy, autoimmune/inflammatory diseases, and beyond.

In the future, by integrating ASE data into donor selection algorithms, “expression-permissive” mismatches (low-expressing alleles) can be prioritized when a fully matched donor is unavailable, which may refine risk stratification. Matching strategies that consider both detailed four-field HLA genotypes and regulatory variants in the promoter and 3′ UTRs could allow for more precise predictions of HLA protein expression on the cell surface and its potential to trigger immune responses. 

## Data Availability

No datasets were generated or analysed during the current study.
